# Commentary: Advocating for patient and public involvement and engagement in health economic evaluation

**DOI:** 10.1186/s40900-023-00444-3

**Published:** 2023-07-03

**Authors:** Sophie Staniszewska, Ivett Jakab, Eric Low, Jean Mossman, Phil Posner, Don Husereau, Richard Stephens, Michael Drummond

**Affiliations:** 1 Warwick Medical School, University of Warwick, Coventry, UK; 2 Patient Policy Research Unit, Syreon Research Institute, Budapest, Hungary; 3 EUPATI Patient Expert, Budapest, Hungary; 4 Eric Low Consulting, Edinburgh, UK; 5 Scotland, UK; 6 Gainesville, FL USA; 7 School of Epidemiology and Public Health, University of Ottawa, Ottawa, Canada; 8 NCRI Advocates Forum, Stevenage, UK; 9 Centre for Health Economics, University of York, York, UK

**Keywords:** Patient and public involvement, Patient and public engagement, Health economic evaluation, CHEERS 2022

## Abstract

**Background:**

Patient and public involvement in health economic evaluation is still relatively rare, compared to other areas of health and social care research. Developing stronger patient and public involvement in health economic evaluation will be important in the future because such evaluations can impact on the treatments and interventions that patients can access in routine care.

**Main text:**

The Consolidated Health Economic Evaluation Reporting Standards (CHEERS) is a reporting guideline for authors publishing health economic evaluations. We established an international group of public contributors who were involved in the update of the CHEERS 2022 reporting guidance, ensuring two items (areas of reporting) specifically about public involvement were included. In this commentary we focus on the development of a guide to support public involvement in reporting, a key suggestion made by the CHEERS 2022 Public Reference Group, who advocated for greater public involvement in health economic evaluation. This need for this guide was identified during the development of CHEERS 2022 when it became apparent that the language of health economic evaluation is complex and not always accessible, creating challenges for meaningful public involvement in key deliberation and discussion. We took the first step to more meaningful dialogue by creating a guide that patient organisations could use to support their members to become more involved in discussions about health economic evaluations.

**Conclusions:**

CHEERS 2022 provides a new direction for health economic evaluation, encouraging researchers to undertake and report their public involvement to build the evidence base for practice and may provide some reassurance to the public that their voice has played a part in evidence development. The CHEERS 2022 guide for patient representatives and patient organisations aims to support that endeavour by enabling deliberative discussions among patient organisations and their members. We recognise it is only a first step and further discussion is needed about the best ways to involve public contributors in health economic evaluation.

**Supplementary Information:**

The online version contains supplementary material available at 10.1186/s40900-023-00444-3.

## Plain language abstract

**Background:**

Patient and public involvement in health economic evaluation is still relatively rare, compared to other areas of health and social care research. Developing stronger patient and public involvement in health economic evaluation will be important in the future because such evaluations can impact on the treatments and interventions that patients can access in routine care.

**Main text:**

We established an international group of public contributors who were involved in the development of the CHEERS 2022 reporting guidance, ensuring two items (areas of reporting) specifically about patient and public involvement were included. In this commentary we focus on the development of a guide to support patient and public involvement in reporting, a key suggestion made by the CHEERS 2022 Public Reference Group, who advocated for greater public involvement in health economic evaluation. The need for this guide was identified during the development of CHEERS 2022 when it became apparent that the language of health economic evaluation is complex and not always accessible, creating challenges for meaningful public involvement in key deliberation and discussion. We took the first step to more meaningful dialogue by creating a guide that patient representatives and patient organisations could use as support to become more involved in discussions about health economic evaluations.

**Conclusions:**

CHEERS 2022 provides a new direction for health economic evaluation, encouraging researchers to undertake and report their public involvement in order to build the evidence base for practice. The CHEERS 2022 guide aims to support patient representatives and patient organisations to become more involved in discussions about health economic evaluations. We recognise it is only a first step and further discussion is needed about the best ways to involve public contributors in health economic evaluation.

**Supplementary Information:**

The online version contains supplementary material available at 10.1186/s40900-023-00444-3.

## Background

Health economic evaluations are comparative analyses of alternative courses of action in terms of their costs and consequences. These evaluations are becoming increasingly influential in decisions about the use of health interventions. For example, the National Institute of Health and Care Excellence (NICE) in England uses the criteria of *‘clinical and cost-effectiveness’* in formulating its recommendations [1]. Such arrangements exist in many other countries that consider both clinical and cost effectiveness in decisions about the provision of care [2]. Therefore, it is possible that a new treatment, while clinically effective, may not be recommended, or may be restricted in its use, because of its cost. As this can impact on whether patients can access treatments, it is vital that patients and the public can understand and be involved with the way such decisions are made, even from a moral perspective. In the future patients and the public should be involved in the refinement or development of the wider concepts, methods and approaches used in health economic evaluation.

To ensure that health economic evaluations are reported consistently, the original Consolidated Health Economic Evaluation Reporting Standards (CHEERS) checklist was developed and published in 2013 [3]. For example, it was intended to help authors accurately report which health interventions were being compared and in what context, how the evaluation was undertaken, what the findings were, and other details that may help readers in understanding and using an evaluation. Since the publication of the original CHEERS checklist, patient and public involvement and engagement (PPIE), sometimes shortened to public involvement, has grown internationally in health and social care research, although it remains relatively rare in health economics, with some exceptions such as the development of frameworks for PPIE in mathematical and economic modelling [4]. However, economics as a discipline has not evolved with integral patient and public in the development of its concepts and methods to the same extent as other areas of health and social care research.

More recently we have seen early signs of more active forms of patient collaboration in the conduct of economic evaluations, but there is still a long way to go and our ambition is that the CHEERS checklist will support more active forms of collaboration, rather than only including patients as sources of data [5–9].

In planning for the update of CHEERS, the ISPOR Task Force recognised that the increasing role of patients and the public in research needed to be reflected in health economic evaluation reporting, and by implication in the conduct of the health economic evaluation [8, 9]. This commentary reports on the public involvement in the development of CHEERS 2022, particularly focusing on the establishment of our international PPIE Reference Group who identified the need to develop guidance for patient representatives and patient organisations and the glossary of terms (Additional file 1: Appendix 1) so they can become more involved in health economic evaluation. All members of the Public Reference Group are co-authors.

### Patient and public involvement

We recognise that involvement in health economic evaluation needs to recognise that input can be different from a patient (in general terms), a public contributor or a patient with lived experience of a condition. In this paper we use the term public contributors to describe individuals who were involved in the development of CHEERS and who had a wider public perspective. They were not primarily involved because of their lived experience but as in other contexts sometimes public contributors can draw on wider lived experience, either their own or that of others they may know or represent.

### Establishing an international Public Reference Group

We established an international Public Reference Group to guide public involvement in the development of CHEERS 2022 with the intention of embedding public involvement at key stages of CHEERS checklist update. Our Public Reference Group included individuals with an interest in the reporting of health economic evaluation, who have knowledge of research and health technology assessment (which routinely uses health economic evaluation) and had been involved in a range of studies and initiatives. We selected individuals who would represent a public view, rather than a specific area of patient experience, as we recognised that the discussion about CHEERS items would happen at a macro level, rather than focusing on specific areas of patient or lived experience.

### Public involvement in the CHEERS 2022 process

The CHEERS checklist was updated through multiple rounds of surveys aiming to reach consensus (a modified Delphi Panel exercise) where experts in economic evaluation, as well as those with perspectives in journal editing, decision making, health technology assessment, and commercial life sciences were invited to participate [8, 9]. Besides invited to the Delphi Panel, the Public Reference Group were able to provide input through multiple meetings at strategic time points of the process. We arranged a series of meetings or ‘knowledge spaces’ virtually using the Microsoft Teams programme that provides a facility for online meetings, to create opportunities for deliberative dialogue about CHEERS [4]. These meetings included Task Force members presenting on the background and development of CHEERS. In the first meeting we reviewed the CHEERS items with public contributors commenting on item wording and meaning. Each item in the CHEERS checklist was considered separately. The Task Force then edited the items, drafted new items and circulated them to the Public Reference Group. These items were then discussed at the second meeting, the Delphi exercise, ensuring PPIE was built in early in the process. In meeting three the focus was on reviewing progress, developing ideas for resources to support patient and public in dissemination of CHEERS which informed the guide included as an appendix. The draft paper, the document supporting PPIE in health economic evaluation and final checklist was sent to the Public Reference Group for comment and input which was acted on. Each item was then reviewed by the Public Reference Group and discussed with some editing to clarify meaning from a public perspective. The Public Reference Group identified the need for additional items (areas of reporting) to capture any patient and public involvement in a health economic evaluation. After discussion with the Public Reference Group and with wider collaborators and following a process of editing and refinement, two key items were included in the checklist. These items are Items 21 and 25 in the CHEERS 2022 checklist, see Table 1. To report on PPIE in the update of the CHEERS checklist, a completed Guidance for Reporting Involvement of Patients and the Public-Version 2 (GRIPP2) checklist was published together with the CHEERS 2022 update [10].

**Table 1:** PPIE items and explanations in CHEERS 2022

*Item 21*
Approach to engagement with patients and others affected by the study: Describe any approaches to engage patients or service recipients, the general public, communities, or stakeholders (eg, clinicians or payers) in the design of the study
*Explanation*
PPIE, wider community engagement, and stakeholder involvement aim to enhance the relevance, acceptability, and appropriateness of research, ultimately improving its quality
Community engagement directly involves local populations in all aspects of decision making, implementation, and policy. It can strengthen local capacities, community structures, and local ownership to improve transparency, accountability, and optimal resource allocations across diverse settings. To understand the contribution PPIE or community engagement makes to research, we encourage reporting of the approach to stakeholder and PPIE when included in health economic evaluation
Acknowledging that PPIE and community engagement in health economic evaluation is still in its infancy, this item requires authors to report any approaches they use and is purposively broad. In addition to reporting the general approach to PPIE, authors may wish to report more specific details of PPIE using GRIPP2 guidance
*Item 25*
Effect of engagement with patients and others affected by the study: Report on any difference patient/service recipient, general public, community, or stakeholder involvement made to the approach or findings of the study
*Explanation*
A key area of reporting is the difference, or the impact, patient, public, community, and stakeholder involvement has made to research because this builds the evidence base for practice
When studies have involved patients, carer, payers, the public, or communities as active collaborators in the research process (as opposed to participants in a research study), we would encourage authors to report any difference this involvement made to the research. Differences may include differences in scope, methods, results, interpretation of results, or process of research. In addition to reporting the difference or impact of public or stakeholder involvement, authors may wish to report more detailed aspects of PPIE using GRIPP2 guidance [10]

### Creating a guide for patient representatives and patient organisations

In addition to discussions about the items, the Public Reference Group also discussed the need to create resources that support patients and public contributors to engage in discussions about health economic evaluation. In response to this we developed a guide to support patient and public involvement in health economic evaluation, which the Public Reference Group commented on, resulting in further refinements. For example, they suggested we include all items rather than a selection of indicative items which was the original intent, to provide a steppingstone into the world of health economic evaluation.

### A second layer of feedback

The guide underwent a further round of peer review with six public contributors who had not been involved in the process so far and one chair of an international Public Reference Group familiar with HTA. Despite our attempts to simplify language it was still felt that the language and concepts were complex and that is would be better if the Guide was targeted at patient representatives and organisations who already have some knowledge or already input into health economic evaluations. They would then be able to support their members to develop the knowledge required to contribute. We have provided a glossary of terms to help develop understanding. We recognise that a further layer of translation and adaptation is required to create a truly patient friendly resource for individual patients or public contributors who are completely unfamiliar with health economic evaluation, and we hope to achieve that in the future. See Additional file 1: Appendix 1 for the Understanding and Interpreting Economic Evaluations in Health Care—A Guide for Patient Representatives and Organisations.

### A call for more integrated patient and public involvement in health economic evaluation

Our effort to create a guide for patient organisations and patient representatives reflects our perspective that patient and public involvement needs to become a routine part of health economic evaluation. Patient and public involvement has become an embedded activity in health and social care research and is common internationally in health technology assessment. The same progress has not been seen in health economic evaluation. We hope the publication of CHEERS 2022 will introduce new impetus into active forms of collaboration between patients and health economics. This will not be represented by patients as subjects in studies providing data, for example, indicating their preferences. Rather it will be represented by active forms of involvement and partnership, with health economic evaluations being carried out ‘*with’ or ‘by’* members of the public rather than ‘to’, ‘about’ or ‘for’ them [11]. In addition to the content of evaluations, we would also encourage the world of health economics to consider how patients and public contributors are involved in the future development of concepts and methods, to reflect the reality of patients’ lives more appropriately in the ways in which health economic evaluation is conducted. Such public involvement in conceptual and methodological aspects of health economic evaluation could represent a new dawn of collaboration, placing patients as partners at the heart of health economic evaluation.

## Discussion and conclusion

CHEERS 2022 is the first health evaluation reporting guideline that has included two items on patient and public involvement and engagement. We recognise that it only represents a starting point and as with all guidance, will evolve over time as the evidence base supporting patient and public involvement in health economic evaluation develops and strengthens. It also represents a nudge to the health economic evaluation community to consider the potential for more embedded forms of involvement, so they can confidently report items 21 and 25. For more complex PPIE reporting, GRIPP2 can be used to support CHEERS 2022 [10]. Our ambition is that by creating the guide for patient organisations we hope to encourage more dialogue about health economic evaluation and more discussion about the concepts, methods and assumptions that are important in such deliberation. We believe that patients and the public can make important contributions to health economic thinking and our ambition is that CHEERS 2022 forms the start of a larger endeavour which sees public involvement as the norm, not the exception, in future health economic evaluation.

## Supplementary Information

**Additional file 1.** Understanding and Interpreting Economic Evaluations in Healthcare: A Guide for Patient Representatives and Organisations.

## Data Availability

Not relevant.

## Declarations

### Authors information

Jean Mossman, Phil Posner and Richard Stephens are patient authors.
