# Supplementary material for: Commentary: Advocating for patient and public involvement and engagement in health economic evaluation
Source: Res Involv Engagem. 2023 Jul 3;9:45. doi: 10.1186/s40900-023-00444-3 (PMC10316557; doi:10.1186/s40900-023-00444-3)
Supplement: Supplementary file 1 — Additional file 1. Understanding and Interpreting Economic Evaluations in Healthcare: A Guide for Patient Representatives and Organisations. [file 40900_2023_444_MOESM1_ESM.docx]

**UNDERSTANDING AND INTERPRETING ECONOMIC EVALUATIONS IN HEALTHCARE**

**A Guide for Patient Representatives and Organisations**

**Sophie Staniszewska^1^**

**Ivett Jakab^2^**

**Eric Low^3^**

**Jean Mossman^4^**

**Phil Posner^5^**

**Don Husereau^6^**

**Michael Drummond^7^**

**Richard Stephens ^8^**

**1. Warwick Medical School, University of Warwick, UK**

**2. Patient Policy Research Unit, Syreon Research Institute, Budapest, Hungary and EUPATI Patient Expert, Budapest, Hungary**

**3. Director, Eric Low Consulting, Edinburgh, UK**

**4. Patient Representative, Scotland, UK**

**5. Patient Representative, Gainesville FL, USA**

**6. School of Epidemiology and Public Health, University of Ottawa, Canada**

**7. Centre for Health Economics, University of York, UK.**

**8. Patient Representative, Stevenage, UK.**

**The terms highlighted in yellow are explained in the glossary.**

| **I. Why is it important for patients and the public to understand economic evaluations in health care?**  The importance of understanding clinical evaluations of health interventions (i.e. health treatments, technologies and programmes) is clear to patients and the public. But it is also necessary for patients and the public to understand and interpret economic evaluations, research studies that assess whether the extra benefits of a treatment or a programme justify any extra costs, when compared with the current standard of care.  There are two main reasons. First, economic evaluations are becoming more influential in decisions about the use of health interventions. For example, the National Institute of Health and Care Excellence (NICE) in England uses the criteria of *‘clinical and cost-effectiveness’* in formulating its recommendations to the National Health Service (NHS). Similar arrangements exist in many other countries. Therefore, it is possible that a new treatment, while clinically effective, may not be recommended, or may be restricted in its use, because of its cost. Since this can affect patients’ access, it is critical that patients and the public understand the basis on which these decisions are made. Such an understanding would also help patients to become more actively involved in the development of economic evaluations and, in time, support patient involvement in the development of key concepts and methods that enable economic evaluations to be more patient-focused and in the public interest.  Secondly, the costs assessed in economic evaluations represent more than mere financial amounts. In a resource-constrained health care system, like many publicly funded ones, the health care costs represent services for other patients that may be displaced. That is, if the budget is fixed, adoption of a new treatment by the health care system means that other services may be discontinued to make way for it. (Economists call this the ‘opportunity cost’.) Therefore, it is in the interests of patients and the public that health care resources are used efficiently and that the various possible trade-offs in the use of resources are adequately explored. It is also important that patients can contribute to discussions about which costs should be included in the evaluation, particularly if there is a wider patient or public cost not being considered.  The views of patients and the public are also important in discussions about whether the health care budget is set at an appropriate level in publicly funded health care systems, since if the budget were increased the opportunity cost of providing a new service would change, as fewer existing services would need to be displaced.  The main audiences for this guide are patient representatives and organisations wishing to increase their understanding of economic evaluations. In several countries these parties are involved in decisions about the adoption of health interventions, through their membership of expert committees of health technology assessment bodies, or through commenting on draft reports produced by these bodies. It is possible that the guide may be useful for individual patients, their carers or members of the public, providing they have some background knowledge of health care. However, for most individual patients a more general introduction would be needed. To this end we are producing a plain language summary of the main points made in the guide.  **2. Improving the reporting of economic evaluations**  The first step in enabling patients and the public to understand economic evaluations is to improve the quality of reporting of the methods used and the results obtained. This was the aim of the Consolidated Health Economic Evaluation Reporting Standards (CHEERS) initiative^1^. The main audiences of CHEERS are the authors of economic evaluations and the journal editors that peer review them. However, recognising the growing role of patients and the public in health care resource allocation decisions, the researchers updating the CHEERS recommendations established a patient and public involvement and engagement group (Public Reference Group) to provide input on the updated version. (See the Box). | **Clinical evaluation**  A study comparing two or more health care interventions in terms of whether they do more good than harm.  **Health intervention**  Any action taken to improve the health of individuals or the population. Typically a treatment or use of a health technology to improve the health of an individual, but could also include preventive measures or improvements in the organization of health care.  **Economic evaluation**  A study comparing two or more health interventions in terms of their costs and consequences. Consequences could include health outcomes or reduced costs in other health care interventions. For example, a vaccination programme against a particular disease has costs in giving the vaccine but may save costs through reducing the occurrence or severity of cases of the disease.  **Cost-effectiveness**  An assessment of whether the additional benefits from using a health intervention justify its extra costs. To be ‘cost-effective’, the benefits from a health intervention need to be greater than its (opportunity) cost.  **Opportunity cost**  The value of a given resource in its best alternative use. To economists this is the value that we forgo when we use a resource in a particular treatment. To be ‘cost-effective’ the benefits from giving the treatment need to be greater that the opportunity cost incurred.  **Health technology assessment**  HTA is a multidisciplinary process that uses explicit methods to determine the value of a health technology at different points in its lifecycle. The purpose is to inform decision making in order to promote an equitable, efficient, and high-quality health system.  . |
| --- | --- |

***The Public Reference Group in the CHEERS Update***

*The Public Reference Group was made up of individuals with an interest in the reporting of health economic evaluations, who have knowledge of research and health technology assessment (HTA) and have been involved in a range of studies. Individuals were also selected who would represent a public view, rather than a specific area of patient experience, since the discussion about CHEERS reporting items was conducted from a broad, health care perspective, rather than focusing on specific areas of patient experience. The Group was moderated by Sophie Staniszewska and briefed on aspects of the CHEERS checklist by the CHEERS co-chairs, Don Husereau and Michael Drummond. Together they form the authorship of this paper.*

*The Public Reference Group used a series of three meetings or ‘knowledge spaces’ to create opportunities for deliberative dialogue about CHEERS. These meetings included the CHEERS co-chairs presenting on the background and development of CHEERS. In the first meeting the Group reviewed the CHEERS items with public contributors commenting on item wording and meaning. Each item was considered separately. The CHEERS team then edited the items, drafted new PPIE items and circulated that to the Public Reference Group. These items were then discussed at the second meeting, prior to the wider circulation of the checklist to a broader group of researchers and journal editors, thereby ensuring PPIE was incorporated early in the process. In meeting three the focus was on reviewing progress, developing ideas for resources to support patient and public dissemination of CHEERS. The draft paper and final checklist was sent to the Public Reference group for comment. In between meetings, the Group commented on further versions of the Guidance for Reporting Involvement of Patients and the Public (GRIPP)^2^ document.*

| **3. What to look for in an economic evaluation**  The full CHEERS 2022 checklist^3^ (shown in Table 1) consists of 28 reporting items, which the authors of published papers about the results of economic evaluations are expected to follow. The checklist is endorsed by several major health services journals, which have indicated a preference for authors to follow the reporting guidelines, although compliance with CHEERS by study authors is voluntary. In using the CHEERS checklist, authors are asked indicate where, in their paper, each reporting item is addressed, so that readers can easily assess whether the report is adequate.  Here we discuss the 28 items, outlining the rationale for making sure they are adequately reported, and stressing the particular issues that patients and the public may wish to consider.  *Title and Abstract*  The first reporting items are the Study Title and Abstract. The CHEERS guidelines state that the title should identify the study as an economic evaluation and the interventions (treatments) being compared. In addition, in common with most journal guidelines, the abstract should provide a structured summary that highlights the economic evaluation context, key methods, results and alternative analyses. **In principle, the title and abstract should give you a good indication of the content of the economic evaluation and help you to decide whether or not the read the full-text article.** However, where journals impose a strict word limit on the abstract, sufficient detail may not be given.  *Background and objectives (of the study)*  It is useful to understand why a given study was conducted and, if relevant, the specific policy or economic question being addressed. In particular, it is useful to know whether the study was being undertaken to help a health technology assessment agency or health care decision maker determine whether a particular therapy should be recommended, as this could directly affect patient access to care. **Your perspective of the importance of the question being addressed may make this the best point at which to decide whether to read further.**  *Health economic analysis plan*  It is considered to be good practice in research to set out the analysis plan for a study in advance. This is intended to reduce the potential for biases being introduced by researchers exploring a number of methods or approaches, and selecting the one that generates the set of results that they prefer (eg the approach that shows the new treatment to be highly cost-effective). **If an analysis plan does exist,** **you can compare the original objectives with the methods used and outcomes reported. Differences between the two may imply potential bias and could be a subject of questions addressed to authors of the study report.** Analysis plans are now commonplace in clinical evaluations, but less so in economic evaluations. Therefore, the CHEERS group felt that economic researchers should be encouraged to say whether an analysis plan existed and where it can be found, as this would increase the general level of confidence and trust in economic evaluations.  *Study population*  As is the case with clinical evaluations, it is important to know precisely what patient population the study applies to. This is typically more specific than a particular health condition (eg multiple myeloma or non-small-cell lung cancer) and will often be defined in terms of a sub-set of patients based on stage of disease (eg newly-diagnosed, relapsed or refractory myeloma), or genetic identification (eg ALK-positive, EGFR-positive or BRAF mutation-positive non-small-cell lung cancer). **If the study results are intended to serve as a basis for a public health-care decision, you can compare the target population of the study versus the decision context, as it might be broader or more narrow. Any difference may be worth discussing with decision-makers.**  *Setting and location*  Whereas the results of clinical evaluations are often generalisable from one setting to another, this is not necessarily the case with the results of economic evaluations. A therapy that is cost-effective in one location may not be cost-effective in another, due to differences in the prices of resources or differences in patterns of care. **Therefore, it is important to consider whether there are any key differences between the setting where the study was conducted and the one that you are interested in.**  *Comparators*  The cost-effectiveness of a therapy is always assessed compared with that of an alternative. **Therefore, it is important to note the comparator treatment(s) to the therapy of interest in the study and whether these are the same as or similar to those used in your own setting.** This is particularly important if the study was conducted in another country, as the available options and the current standard of care can vary greatly from those available in your own setting.  *Perspective*  Many economic evaluations are conducted from the perspective of the health care decision-maker and therefore restrict their interest to health care costs. **It may be worthwhile considering whether there are important costs or burdens falling on patients and their families that should be considered.** These could include, for example, the provision of informal nursing care, the costs of time and travel required to access the treatment, or time lost from work by patients or those caring for them. There may also be impacts on carers’ quality of life due to the additional burdens they face.  *Time horizon and discount rate*  Economic evaluations consider the costs and outcomes of therapies over a particular period (known as the time horizon). In some studies this may be for the lifetime of a patient, but often it may be shorter (eg based on the length of follow-up in the clinical trials of the therapy concerned). **It is important to consider whether the time horizon of the study is long enough to capture the full benefits or potential harms of the therapy.**  The other key issue to note is the discount rate used. Discounting is an approach used by economists to reflect whether costs and benefits occur in the short-term or the longer term. The effect of discounting is to give a lower importance to costs and benefits occurring in future years. This may seem a rather technical point, but it can have a profound impact on the cost-effectiveness of therapies where the benefits stretch far into the future. Examples include preventive measures, therapies for children, or therapies of curative intent such as gene therapies.  A typical discount rate applied in economic evaluations is around 3% per annum, but sometimes it may be higher or lower. The important point to note is that the higher the discount rate, the lower the value being placed on long-term outcomes, or benefits in the future. This is a particular issue for gene therapies, which often have a high up-front cost, but potentially deliver benefits over a lifetime. Therefore, the cost-effectiveness of these therapies varies considerably depending on whether we apply a discount rate of 1,3, or 5% per annum.  In most jurisdictions the rate by which we chose to discount the future in public investments is determined by the Ministry of Finance, based on its judgment of the extent to which current benefits should be forgone in favour of benefits in the future. In making this judgment it is common to take account of the interest rate on low-risk investments, such as the rate on long-term government bonds. However, there may be occasions where the impact of discounting should be discussed or questioned. Sometimes those conducting studies report the cost-effectiveness of the therapies being compared using different discount rates, which can facilitate this discussion. **You may question whether the effect of using different discount rates has been adequately investigated, especially for interventions benefiting the patient over a long time horizon.**  *Selection, measurement, and valuation of outcomes*  This is probably the issue in which economic evaluations would benefit most from patient and public involvement and it would be worth checking whether this involvement was sought. The outcomes measured in clinical studies may not be those of most relevance to patients. For example, the outcomes measured might be surrogate endpoints, which are included in clinical studies to show that the treatment (typically a drug) has some activity. However, they may have little relevance to the patient’s condition unless they are associated with changes in quality of life, or good predictors of final outcomes.  Economic evaluations typically consider the gains in increased survival and improved quality of life resulting from health interventions. Some other items of interest to patients, such as the convenience of using the various therapies, due to the treatment being easier to take or being more easily available locally, may not be considered unless the greater convenience results in a higher adherence to the therapy and hence improved effectiveness.  Economic evaluations often consider various patient-reported outcome measures (PROMs) of quality of life, but the outcome most favoured by economists is a measure of overall gain in length and quality of life, known as he quality-adjusted life-year or QALY. The QALYs gained from a health intervention are calculated by multiplying the number of life years gained by the value of the health states that the individual experiences over those years.  While QALYs have their merits, it is important to explore how these were calculated in the economic evaluation. **A key issue is the determination of the value of health states, often known as ‘health utilities’. It is important to know whose preferences were used to determine the health utilities** (eg Were these the preferences of the patients being treated or those of the public more generally?). This may be particularly important if it is thought that preferences may differ substantially from one group to another. For example, individuals with a health condition may be willing to bear a greater risk of adverse effects in order to receive therapy than would members of the general public.  Most of the widely used instruments to measure and value health states, such as the EQ-5D^4^ rely primarily on preferences from the public. Measurement instruments like the EQ-5D are preferred by health technology assessment agencies, like NICE in the UK, that make recommendations on the use of treatments. These ‘generic’ instruments have the advantage that they can be used across all fields on medicine, but may not always reflect patient experience, or detect small improvements in quality of life that may be important to patients such as the convenience of dosing or pill size.  **You may question the relevance of chosen clinical outcomes and patient-reported outcome measurements (PROMs) (eg generic vs. disease-specific) for the target patient population, or whether the PROMs have been validated for use in the specific patient population.**  *Measurement and valuation of resources and costs*  Although patients and the public are likely to be primarily interested in the selection, measurement and valuation of outcomes, the measurement and valuation of resources and costs is equally important for determining whether the health intervention of interest is cost-effective. **The first thing to check is whether the range of resources/costs considered is consistent with the perspective adopted in the study and whether there is an omission of any items that would be of relevance to the patient and public.** For example, a study undertaken from a health care system perspective may not include consideration of any of the costs patients incur in accessing treatment.  Secondly, it is important that the measurement of quantities of resources (eg number of hospital admissions, or days of hospital stay) is reported separately from the sources of valuation, since this is critical to an assessment of whether the costs in the location where the evaluation was undertaken are typical of those in your setting. For example, if the authors report separately the number of days of hospitalization and the cost of a day in hospital, it may be possible to obtain equivalent information from your own setting. The cost of a day spent in the hospital can be substantially different across countries or locations, whereas the number of days spent in the hospital with a specific treatment might be more constant). **Considering separately the quantity of a resource items used and the cost per item, might help you in assessing the limitations of adopting the study results to your local context.**  *Currency price date and conversion*  If the values of resources are to be compared, they need to be in the same currency and, given the possibility of inflation, the same price date (eg 2022 US dollars). **Therefore, it is important to check that these conversions have been made**, although failure to do so does not systematically bias the estimates in favour or against the interests of patients.  *Rationale and description of the model*  Many economic evaluations employ/use an economic model to synthesize data on treatment effectiveness and cost, and/or to project costs and benefits in the long term. The model, which should be clearly described in the study, determines the clinical pathways being explored. This can be represented in a flow diagram showing how patients move through different treatment pathways and the outcomes associated with each pathway. **Therefore, it is important to assess whether the clinical pathways used in the model are realistic, and whether they truly reflect the patient pathway**. This is another element of economic evaluations that would benefit from patient and public involvement, as well as that of health professionals. Patients can have a role in determining which variables contribute to the model as well as wider contextual factors that might impact interpretation of the results. **Patients can also challenge the limitations of the model (eg the clinical pathways considered and uncertainties around the data (eg the use of assumptions where data do not exist).**  *Analytics and assumptions*  Given the large number of estimates of costs and outcomes in an economic evaluation, it is unlikely that all of these will be precise. Sometimes, information is not available for some items and the authors of the study may have to rely on assumptions, made either by themselves or clinical experts. **It is important that these assumptions are made transparent and that the logic behind them is explained. This can help you to question the approach that has been followed.**  In addition, where assumptions are made, ideally the authors will explore whether these have a major impact on the results of the evaluation. This process is called ‘sensitivity analysis’.  *Characterizing heterogeneity*  Heterogeneity refers to the possible differences in costs or outcomes for different patients, or patient sub-groups receiving the therapies being compared. This may relate to differences in the seriousness of the patients’ illness prior to treatment (known as ‘baseline risk’) and differences in treatment response. This is important in clinical studies but becomes even more critical in economic evaluations, especially if these are being used to formulate recommendations for access to therapy. For example, a new therapy may be cost-effective for some sub-groups of patients but not others affected by the same disease. In turn this may lead to restrictions in patient access. **Therefore, it is important to consider whether the study explores heterogeneity among patient sub-groups (ie whether the same cost-effectiveness conclusions apply for all sub-groups) and whether this is done appropriately.** Even though cost-effectiveness is higher in some patient groups than others, it still may be justifiable to give access to all^5^.  *Characterizing distributional effects*  This item is linked to the previous one, as it involves the exploration of any inequalities between individuals or groups in the study, whether due to clinical circumstances or the impact of socio-economic factors. For example, a new vaccine might be very cost-effective overall, but not taken up by people with low incomes, or certain ethnic groups, perhaps because of the difficulties of traveling to health care facilities or lack of awareness. **Therefore, it is important to explore whether adopting a particular policy would not be advantageous for some groups and whether this should be taken account of in the decision.** For example, adopting a therapy that is slightly less cost-effective overall may allow greater access to some sub-sets of the patient population. For example, a service that is only available in specialist centres may be difficult to access for some individuals living further away from these centres.  *Study parameters; Summary of main results; Characterizing uncertainty; Effect of uncertainty*  Several issues are important here. As well as presenting the overall summary study result, often in the form of the incremental cost per QALY gained, it is useful if the results are presented in a disaggregated manner; eg the life years gained, QALYs gained and costs of the health intervention of interest and the comparator. It may also be helpful if these estimates are reported in an undiscounted and discounted form, so that the impact of the discount rate can be assessed. Finally, as mentioned in the discussion of Item 17 above, where estimates are not precise or assumptions have been made, it important to characterise this uncertainty by undertaking a sensitivity analysis, varying estimates to determine how much difference this makes to the study results. **It is important to check whether a sensitivity analysis was conducted, and if it was, which items of the study affect the results the most. It may be worth questioning the reliability and robustness of data sources for the most influential items, and whether obtaining better quality data could impact the conclusions of the study.**  *Study findings, limitations, generalizability and current knowledge)*  In principle, a considerable amount of information could be reported under this item. The main issues to consider are (i) whether the limitations in the study are clearly acknowledged (ii) whether a particular decision rule (eg a cost per QALY threshold) is being applied in determining whether the therapy of interest is ‘cost-effective’ and (iii) whether other important considerations, that were not assessed as part of the study, are discussed. **Limitations are inevitable in conducting any economic evaluation. However, if these are stated clearly, you can question them and assess the results accordingly.**  *Approach to, and effects of, patient, public, community and stakeholder involvement and engagement*  As a result of the PPIE in the development of the CHEERS 2022 checklist, two items were added to encourage authors to report any efforts to engage patients, the public, communities, and other stakeholders in the study and, if so, what impact it had. **These items provide an opportunity for researchers and patients to highlight any public involvement in their economic evaluation and allow you to question the approach used.** We also hope they will encourage those designing and conducting health economic evaluations to more actively involve patients in the conceptualisation and undertaking of these studies.  *Sources of funding and Conflicts of interest*  The declaration of sources of funding and conflicts of interest is standard practice in all evaluations in the health care field. The existence of conflicts of interest does not automatically imply bias, but they should be declared so that the research can be subjected to appropriate scrutiny. For example, if the cost-effectiveness of a new drug has been investigated by researchers receiving funding from the drug’s manufacturer, the conclusions should be handled with extra caution until a detailed critical appraisal of the evaluation has been made by an independent party. **You should pay close attention to potential biases that may arise from the sources of funding of the study and conflicts of interest of authors.**  **4. Taking matters forward**  The objective of producing this guide was to enable patient representatives and organisations to be more involved in the conduct and use of economic evaluations in health care. Economic evaluations are likely to be more influential in decisions whether or not to allow the use of health interventions in the future, as the pressures on health care budgets grow. Therefore, it is important that the voice of patients and the public is heard in these discussions.  We hope that reading this guide will stimulate patients and the public to be more involved in economic evaluations in various ways, including advising researchers as they develop their studies, and being members of committees within health technology assessment agencies that use the results of economic evaluations to make recommendations on the use of health interventions in health care systems.  Patients and patient organizations should feel free to use this guide, in its entirety or in part, to develop their skills in critiquing completed economic evaluations, in participating in the development of future evaluations, and in educating others about economic evaluation. In addition to this guide, those with a deeper interest could read the full CHEERS report^3^, or consult the summaries of existing economic evaluations on the Tufts Cost-Effectiveness Registry or the Global Health Cost-Effectiveness Registry^6,7^. | **Journal guidelines**  The guidelines academic journals give authors considering writing and submitting a paper. They often prescribe how papers should be formulated, or how declarations of conflicts of interest should be made.  **Health technology assessment agency**  An organization that makes recommendations on the use of health technologies (health interventions) to health care decision makers.  **Health care decision maker**  A person who makes decisions on how resources are used in the health care system (eg clinicians, administrators, politicians).  **Clinical evaluation**  A study comparing two or more health care interventions in terms of whether they do more good than harm.  **Comparator treatment(s)**  The treatment(s) or health intervention(s) being compared to the treatment of intervention of interest. Typically the comparator to a new treatment would be the existing standard of care.  **Health care costs**  Costs incurred by the health care system (eg costs of hospital care, outpatient care and community care)  **Time horizon**  The period of time over which measurements of outcomes are made in a clinical or economic evaluation. It could be the length of follow-up in a clinical trial, or lifetime in an economic model. The important consideration is that the time horizon is long enough to enable measurement of all the clinical or economic outcomes in the evaluation that is being conducted.  **Discount rate**  The annual rate (or percentage) by which costs or benefits occurring in future years are reduced in an economic evaluation.  **Preventive measures**  Health interventions to prevent illness, such as screening or vaccination programmes, or measures to influence individuals’ lifestyles.  **Gene therapies**  Therapies that work by either modifying or replacing the defective gene that causes the disease.  **Outcomes**  Any of the effects or consequences of health interventions, but typically used to denote the clinical effects, such as changes in survival or adverse effects of treatment.  **Surrogate endpoints**  An intermediate endpoint, which is judged to be predictive of the final outcome. For example, in a cancer clinical evaluation time to progression (of the disease) may be predictive of overall survival. Sometimes a biomarker may be a surrogate, such as blood glucose level for diabetes or blood pressure for stroke.  **Final outcome**  An endpoint reflecting the main objective of treatment, such as an improvement in survival or quality of life.  **Patient reported outcome (measures)**  Any outcome measured by asking patients about the impact of a health intervention. Most of these measures relate to aspects of the patient’s quality of life before, during and following treatment.  **Quality-adjusted life-year (QALY)**  A year of life adjusted by the health utility the person is experiencing. It can vary between 0 and 1 (a year lived at full health). Health interventions have the potential to increase a persons’ length of life and/or the quality of their life. The QALYs gained from a health intervention is the difference between the person’s health state and length of life with and without the intervention.  **Health utilities**  The values individuals place on health states, typically presented on a scale between 1 (full health) and 0 (dead).  **Health state**  A person’s level of health, typically defined in terms of their physical, mental and social wellbeing.  **Economic model**  A framework used by economists to synthesise evidence on all the relevant clinical and economic outcomes and costs when undertaking an economic evaluation. Models typically depict the clinical pathways of the health interventions being compared and patients’ progressions though health states over time.  **Clinical pathway**  A sequence of clinical decisions and outcomes that might be experienced by people being treated for a given disease or health condition.  **Sensitivity analysis**  The process by which the impact of uncertainty in estimates is explored in an economic evaluation. Estimates are varied either one at a time (deterministic sensitivity analysis) or simultaneously (probabilistic sensitivity analysis). The advantage of probabilistic sensitivity analysis is that it characterizes the overall uncertainty in the evaluation.  **Heterogeneity**  The variation we observe between sub-groups of the patient population in terms of their baseline risk or treatment response.  **Patient sub-group**  A sub-set of the whole patient population, often defined by baseline risk or treatment response.  **Baseline risk**  A measure of the severity of the disease in a given patient or the population at large.  **Treatment response**  The amount of change in outcome observed following treatment.  **Distributional effects**  The effects or outcomes of a health intervention experienced by different groups of the population, typically defined by ethnicity, geographical location or socio-economic factors (ie level of income or wealth).  **Socio-economic factors**  These relate to individuals’ level of income and wealth.  **Cost per QALY**  The cost incurred to generate one quality-adjusted life-year by using a health intervention. The results of studies are often presented as the incremental cost per QALY gained for the health intervention of interest, over the comparator.  **Cost per QALY threshold**  The maximum level of cost per QALY that is deemed ‘acceptable’ by a decision maker in a given setting. |
| --- | --- |

**References**

**1.** Husereau, D., Drummond, M.F., Petrou, S., Carswell, C., Moher, D., Greenberg, D., *et al*. Consolidated health economic evaluation reporting standards (CHEERS) - explanation and elaboration: A report of the ISPOR health economic evaluation publication guidelines good reporting practices task force. *Value in Health* 2013*,* 16(2):231-250.

2. Staniszewska S, Brett J, Simera I, et al. GRIPP2 reporting checklists: tools to improve reporting of patient and public involvement in research. *Res Involv Engagem* 2017;3:13. doi:10.1186/s40900-017-0062-2.

3. Husereau D, Drummond MF, Augustovski F, Briggs AH, Carswell C et al Consolidated

Health Economic Evaluation Reporting Standards 2022 (CHEERS 2022) Explanation and

Elaboration: A Report of the ISPOR CHEERS II Good Practices Task Force. Value in Health 2022;25(i):10-31.

4. EuroQoL Group. EuroQol: a new facility for the measurement of health-related quality of life. Health Policy 1990; 16: 199-208.

5. Rawlins MD, Culyer AJ. National Institute for Clinical Excellence and its value judgments. BMJ 2004; 329:224-7.

6. CEA Registry. Centre for the Evaluation of Value and Risk in Health, Tufts Medical Center, Boston, USA. <https://cevr.tuftsmedicalcenter.org/databases/cea-registry> (Accessed 2 February , 2022)

7. Global Health CEA Registry. Centre for the Evaluation of Value and Risk in Health, Tufts Medical Center, Boston, USA. <http://ghcearegistry.org/ghcearegistry/>. (Accessed 2 February, 2022)

**Acknowledgements**

We are grateful to Jo Mauskopf, Gabrielle Mathews, Ruth Sambrook, Natalie Ealey, Ann Single and Kelly Lenahan for helpful comments on an earlier draft of this guide. However, we are responsible for any remaining errors or omissions.

Table 1 CHEERS 2022 Statement Checklist

| **SECTION /Topic** | **Item** | **Guidance for Reporting** | **Reported in section** |
| --- | --- | --- | --- |
| **TITLE** | | |  |
| Title | 1 | Identify the study as an economic evaluation and specify the interventions being compared. |  |
| **ABSTRACT** | | |  |
| Abstract | 2 | Provide a structured summary that highlights context, key methods, results and alternative analyses. |  |
| **INTRODUCTION** | | |  |
| Background and objectives | 3 | Give the context for the study, the study question and its practical relevance for decision making in policy or practice. |  |
| **METHODS** | | |  |
| Health economic analysis plan | 4 | Indicate whether a health economic analysis plan was developed and where available. |  |
| Study population | 5 | Describe characteristics of the study population (such as age range, demographics, socioeconomic, or clinical characteristics). |  |
| Setting and location | 6 | Provide relevant contextual information that may influence findings. |  |
| Comparators | 7 | Describe the interventions or strategies being compared and why chosen. |  |
| Perspective | 8 | State the perspective(s) adopted by the study and why chosen. |  |
| Time horizon | 9 | State the time horizon for the study and why appropriate. |  |
| Discount rate | 10 | Report the discount rate(s) and reason chosen. |  |
| Selection of outcomes | 11 | Describe what outcomes were used as the measure(s) of benefit(s) and harm(s). |  |
| Measurement of outcomes | 12 | Describe how outcomes used to capture benefit(s) and harm(s) were measured. |  |
| Valuation of outcomes | 13 | Describe the population and methods used to measure and value outcomes. |  |
| Measurement and valuation of resources and costs | 14 | Describe how costs were valued. |  |
| Currency, price date, and conversion | 15 | Report the dates of the estimated resource quantities and unit costs, plus the currency and year of conversion. |  |
| Rationale and description of model | 16 | If modelling is used, describe in detail and why used. Report if the model is publicly available and where it can be accessed. |  |
| Analytics and assumptions | 17 | Describe any methods for analysing or statistically transforming data, any extrapolation methods, and approaches for validating any model used. |  |
| Characterizing heterogeneity | 18 | Describe any methods used for estimating how the results of the study vary for sub-groups. |  |
| Characterizing distributional effects | 19 | Describe how impacts are distributed across different individuals or adjustments made to reflect priority populations. |  |
| Characterizing uncertainty | 20 | Describe methods to characterize any sources of uncertainty in the analysis. |  |
| Approach to engagement with patients and others affected by the study | 21 | Describe any approaches to engage patients or service recipients, the general public, communities, or stakeholders (e.g., clinicians or payers) in the design of the study. |  |
| **RESULTS** | | |  |
| Study parameters | 22 | Report all analytic inputs (e.g., values, ranges, references) including uncertainty or distributional assumptions. |  |
| Summary of main results | 23 | Report the mean values for the main categories of costs and outcomes of interest and summarise them in the most appropriate overall measure. |  |
| Effect of uncertainty | 24 | Describe how uncertainty about analytic judgments, inputs, or projections affect findings. Report the effect of choice of discount rate and time horizon, if applicable. |  |
| Effect of engagement with patients and others affected by the study | 25 | Report on any difference patient/service recipient, general public, community, or stakeholder involvement made to the approach or findings of the study |  |
| **DISCUSSION** | | |  |
| Study findings, limitations, generalizability, and current knowledge | 26 | Report key findings, limitations, ethical or equity considerations not captured, and how these could impact patients, policy, or practice. |  |
| **OTHER RELEVANT INFORMATION** | | | |
| Source of funding | 27 | Describe how the study was funded and any role of the funder in the identification, design, conduct, and reporting of the analysis |  |
| Conflicts of interest | 28 | Report authors conflicts of interest according to journal or International Committee of Medical Journal Editors requirements. |  |

**GLOSSARY OF TERMS (in alphabetical order)**

**Baseline risk**

A measure of the severity of the disease in a given patient or the population at large.

**Clinical evaluation**

A study comparing two or more health care interventions in terms of whether they do more good than harm.

**Clinical pathway**

A sequence of clinical decisions and outcomes that might be experienced by people being treated for a given disease or health condition.

**Comparator treatment(s)**

The treatment(s) or health intervention(s) being compared to the treatment of intervention of interest. Typically the comparator to a new treatment would be the existing standard of care.

**Cost-effectiveness**

An assessment of whether the additional benefits from using a health intervention justify its extra costs. To be ‘cost-effective’, the benefits from a health intervention need to be greater than its (opportunity) cost.

**Cost per QALY**

The cost incurred to generate one quality-adjusted life-year by using a health intervention. The results of studies are often presented as the incremental cost per QALY gained for the health intervention of interest, over the comparator.

QALYs gained from intervention – QALYs gained from comparator

Incremental cost per QALY gained = ---------------------------------------------------------------------------------------

Costs of intervention - Costs of comparator

Basically, this is an assessment of the extra value obtained from adopting a more expensive therapy. The questions is then ‘is it worth it?’

**Cost per QALY threshold**

The maximum level of cost per QALY that is deemed ‘acceptable’ by a decision maker in a given setting.

**Discount rate**

The annual rate (or percentage) by which costs or benefits occurring in future years are reduced in an economic evaluation.

**Distributional effects**

The effects or outcomes of a health intervention experienced by different groups of the population, typically defined by ethnicity, geographical location or socio-economic status (ie level of income or wealth).

**Economic evaluation**

A study comparing two or more health interventions in terms of their costs and consequences. Consequences could include health outcomes or reduced costs in other health care interventions. For example, a vaccination programme against a particular disease has costs in giving the vaccine but may save costs through reducing the occurrence or severity of cases of the disease.

**Economic model**

A framework used by economists to synthesise evidence on all the relevant clinical and economic outcomes and costs when undertaking an economic evaluation. Models typically depict the clinical pathways of the health interventions being compared and patients’ progressions though health states over time.

**Final outcome**

An endpoint reflecting the main objective of treatment, such as an improvement in survival or quality of life.

**Gene therapies**

Therapies that work by either modifying or replacing the defective gene that causes the disease.

**Health care costs**

Costs incurred by the health care system (eg costs of hospital care, outpatient care and community care)

**Health care decision maker**

A person who makes decisions on how resources are used in the health care system (eg clinicians, administrators, politicians).

**Health intervention**

Any action taken to improve the health of individuals or the population. Typically a treatment or use of a health technology to improve the health of an individual, but could also include preventive measures or improvements in the organization of health care.

**Health technology**

Often used to describe a particular component of a person’s care, such as a drug or medical device. Also, used to describe any component health care and is thus synonymous with health intervention.

**Health technology assessment**

A multidisciplinary process that uses explicit methods to determine the value of a health technology at different points in its lifecycle. The purpose is to inform decision making in order to promote an equitable, efficient, and high-quality health system.

**Health technology assessment agency**

An organization that makes recommendations on the use of health technologies (health interventions) to health care decision-makers.

**Health state**

A person’s level of health, typically defined in terms of their physical, mental and social wellbeing.

**Health utilities**

The values individuals place on health states, typically presented on a scale between 1 (full health) and 0 (dead).

**Heterogeneity**

The variation we observe between sub-groups of the patient population in terms of their baseline risk or treatment response.

**Journal guidelines**

The guidelines academic journals give authors considering writing and submitting a paper. They often prescribe how papers should be formulated, or how declarations of conflicts of interest should be made.

**Opportunity cost**

The value of a given resource in its best alternative use. To economists this is the value that we forgo when we use a resource in a particular treatment. To be ‘cost-effective’ the benefits from giving the treatment need to be greater that the opportunity cost incurred.

**Outcomes**

Any of the effects or consequences of health interventions, but typically used to denote the clinical effects, such as changes in survival or adverse effects of treatment.

**Patient reported outcome (measures)**

Any outcome measured by asking patients about the impact of a health intervention. Most of these measures relate to aspects of the patient’s quality of life before, during and following treatment.

**Patient sub-group**

A sub-set of the whole patient population, often defined by baseline risk or treatment response.

**Preventive measures**

Health interventions to prevent illness, such as screening or vaccination programmes, or measures to influence individuals’ lifestyles.

**Quality-adjusted life-year (QALY)**

A year of life adjusted by the health utility the person is experiencing. It can vary between 0 and 1 (a year lived at full health). Health interventions have the potential to increase a persons’ length of life and/or the quality of their life. The QALYs gained from a health intervention is the difference between the person’s health state and length of life with and without the intervention. (This is the difference between the two curves in the diagram below.)

**Sensitivity analysis**

The process by which the impact of uncertainty in estimates is explored in an economic evaluation. Estimates are varied either one at a time (deterministic sensitivity analysis) or simultaneously (probabilistic sensitivity analysis). The advantage of probabilistic sensitivity analysis is that it characterizes the overall uncertainty in the evaluation.

**Socio-economic factors**

These relate to individuals’ level of income and wealth.

**Surrogate endpoints**

An intermediate endpoint, which is judged to be predictive of the final endpoint. For example, in a cancer clinical evaluation time to progression (of the disease) may be predictive of overall survival. Sometimes a biomarker may be a surrogate, such as blood glucose level for diabetes or blood pressure for stroke.

**Time horizon**

The period of time over which measurements of outcomes are made in a clinical or economic evaluation. It could be the length of follow-up in a clinical trial, or lifetime in an economic model. The important consideration is that the time horizon is long enough to enable measurement of all the clinical or economic outcomes in the evaluation that is being conducted.

**Treatment response**

The amount of change in outcome observed following treatment.
